# Granulomatosis with polyangiitis and neurofibromatosis type 1: a fortuitous association? (a case report)

**DOI:** 10.11604/pamj.2021.39.162.30213

**Published:** 2021-07-01

**Authors:** Berriche Olfa, Romdhane Wiem, Arfa Sondess, Chelly Jihene, El Arbi Fatma, Alaya Wafa

**Affiliations:** 1 Department of Endocrinology and Internal Medicine, Tahar Sfar University Hospital of Mahdia, Mahdia, Tunisia,; 2 Faculty of Medicine, University of Monastir, Monastir, Tunisia,; 3 Biochemistry Laboratory, LR12ES05 LR-NAFS Nutrition-Functional Food and Vascular Health, Faculty of Medicine, University of Monastir, Monastir, Tunisia

**Keywords:** Granulomatosis, polyangiitis, neurofibromatosis type 1, vasculitis, case report

## Abstract

Neurofibromatosis type 1 is a common neurocutaneous syndrome, caused by an alteration of the NF-1 gene, which is a tumor suppressor. It has been reported to be associated with different types of benign and malignant tumors but its association to systemic diseases is uncommon and has not been reported previously to be associated with vasculitis particularly granulomatosis with polyangiitis (GPA). We report a case of a 17-year-old male patient, who, during his follow-up for neurofibromatosis type 1, in our outpatient consultation; we objectified hypereosinophilia at 1700 eosinophils/μl without a history of asthma or atopy. He reported a nasal obstruction with epistaxis and no rhinorrhea or pruritus. Physical examination revealed afebrile patient with the lesions of his neurofibromatosis type 1 without cutaneous rash or urticaria. Rhinoscopy didn’t show any lesion. Laboratory tests revealed a normal renal function, negative 24-hour urine protein, and no biological inflammatory syndrome. Immunological tests noted positives cytoplasmic antineutrophil cytoplasmic antibodies, and a slight increase in antinuclear antibodies at 1/180. Extensive infectious research was negative. Computed tomography (CT) of the sinuses revealed a non-obstructive nasal septum deviation with anatomical variations, and a chest scan showed multiple bilateral pulmonary nodules and micronodules. After ruling out the other etiologies, we retained the diagnosis of granulomatosis with polyangiitis according to American College of Rheumatology (ACR) criteria 1990 and we could start early the treatment. To our knowledge, the association between Neurofibromatosis type 1 and vasculitis, particularly GPA, has not been reported previously, which makes our case original and confirms the utility of an extensive lesion assessment during the follow-up.

## Introduction

Neurofibromatis type 1, also known as Von Recklinghausen's disease, is an autosomal dominant disease; it affects multiple organ systems and has a wide spectrum of clinical manifestations, ranging from characteristic cutaneous, «café au lait» macules and multiple neurofibromas to neurological symptoms, skeletal dysplasias and vascular manifestations. Neurofibromatis has an increased risk of developing benign or malignant tumors; but its association with autoimmune diseases has been rarely reported [1]. However, Granulomatosis with polyangiitis (GPA), previously known as Wegener´s granulomatosis, is an inflammatory systemic vasculitis affecting small- and medium-sized blood vessels [2]. Its association to neurofibromatosis type 1 (NF1) has not been reported in the literature. We report a case of a fortuitous discovery of granulomatous with polyangiitis in a 17-year-old patient followed for neurofibromatous type 1.

## Patient and observation

Neurofibromatis type 1, also known as Von Recklinghausen's disease, is an autosomal dominant disease; it affects multiple organ systems and has a wide spectrum of clinical manifestations, ranging from characteristic cutaneous, café-au-lait macules and multiple neurofibromas to neurological symptoms, skeletal dysplasias and vascular manifestations. Neurofibromatis has an increased risk of developing benign or malignant tumors; but its association with autoimmune diseases has been rarely reported [1]. However, Granulomatosis with polyangiitis (GPA), previously known as Wegener´s granulomatosis, is an inflammatory systemic vasculitis affecting small- and medium-sized blood vessels [2]. Its association to NF1 has not been reported in the literature. We report a case of a fortuitous discovery of granulomatous with polyangiitis in a 17-year-old patient followed for neurofibromatous type 1.

**Figure 1:**
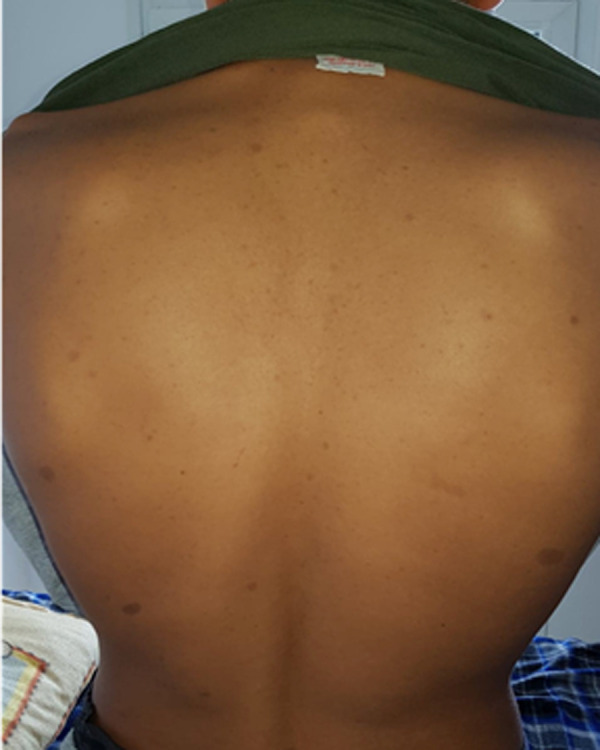
«café-au-lait» macules on the back

**Patient information:** a 17-year-old male patient followed up for neurofibromatosis type 1, diagnosed based on National Institutes of Health (NIH) criteria including multiple café-au-lait spots (Figure 1), fibromas and paternal history. During his follow-up, in our outpatient consultation, we objectified hypereosinophilia at 1700 eosinophils/μl without a history of asthma or atopy. He reported a nasal obstruction with epistaxis and no rhinorrhea or pruritus.

**Clinical findings:** physical examination revealed afebrile patient with lesions of his neurofibromatosis type 1 without cutaneous rash or urticaria. Rhinoscopy didn´t show any lesion.

**Time line:** a 17-year-old male patient followed up for neurofibromatosis type 1 in our outpatient consultation since October 2015; presented hypereosinophilia which was discovered fortuitously in August 2020 and reported then a nasal obstruction with epistaxis.

**Figure 2:**
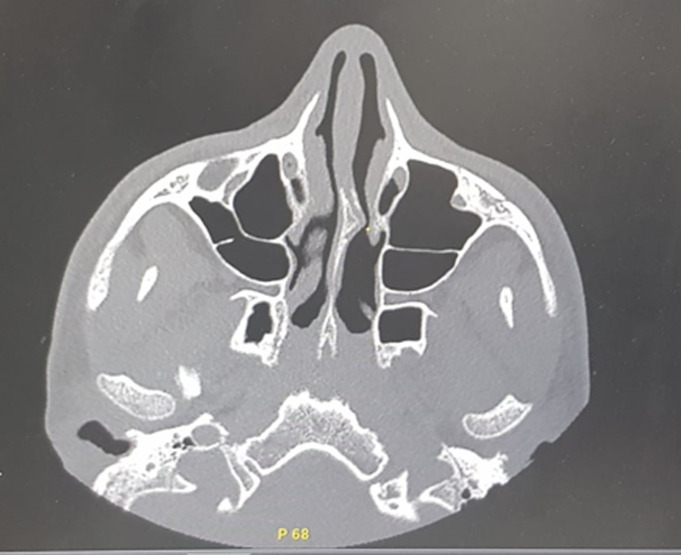
a non-obstructive nasal septum deviation with anatomical variations

**Figure 3:**
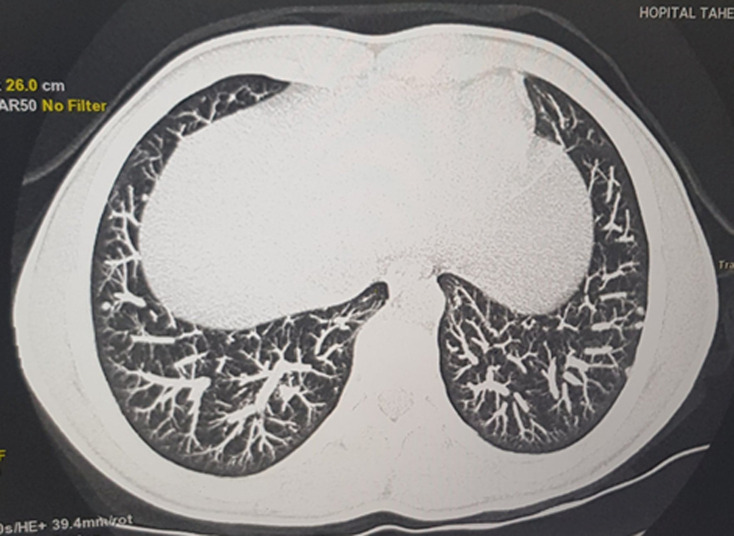
a chest scan revealing pulmonary nodules and micronodules

**Diagnostic assessment:** laboratory tests revealed a normal renal function, negative 24-hour urine protein and no biological inflammatory syndrome. Immunological tests noted positives cytoplasmic antineutrophil cytoplasmic antibodies (ANCA), and a slight increase in antinuclear antibodies at 1/180. Extensive infectious research was negative. Computed tomography (CT) of the sinuses revealed a non-obstructive nasal septum deviation with anatomical variations (Figure 2), and chest scan showed multiple bilateral pulmonary nodules and micronodules (Figure 3). After ruling out the other etiologies, in front to the association to the nasal discharge, epitaxis, the pulmonary involvement and positives cytoplasmic ANCA we retained the diagnosis of granulomatosis with polyangiitis according to American College of Rheumatology (ACR) criteria 1990.

**Therapeutic intervention:** we started an early treatment based on high doses of corticosteroids (1mg/kg/day) with adjuvant therapy and methotrexate (0.2 mg/kg/week).

**Follow-up and outcomes:** while the follow up in the outpatient consultation, after 6 months of starting the treatment, the patient is taking regularly his treatment and described an improvement in nasal obstruction and epistaxis.

**Patient perspective:** the patient is taking regularly his treatment for better prognosis.

**Informed consent:** the patient gave an informed consent.

## Discussion

Neurofibromatosis type 1 is a common neurocutaneous syndrome. It is caused by an alteration of the NF-1 gene, which is a tumor suppressor located on the long arm of chromosome 17 (17q11.2). Loss of this gene's function due to a mutation leads to an increase in cell proliferation and development of tumors [3]. That´s why, NF 1 is significantly associated with an increased risk of developing malignant and benign tumors. However, the association of NF1 and autoimmune diseases has been uncommonly reported. Literature in this subject still limited to case series. In another hand, coexisting NF1 and systemic vasculitis, particularly GPA, was not reported in the literature. Therefore, we ultimately ask the following question: is the association of NF1 to the GPA in our case incidental? In fact NF1 was reported to be associated with Systemic Lupus Erythematosus (SLE), autoimmune hemolytic anemia, autoimmune thyroiditis and vitiligo. Exact pathogenesis leading to theses associations is not well understood; however, authors proposed that the lack of neurofibromin in NF1 led to dysregulation of the RAS pathway and impairment of T cells, creating the autoimmune disorders [1,4]. In another hand, it was well-recognized that a wide spectrum of vascular abnormalities, as aneurysms, stenosis of the aortic, renal, and mesenteric circulation, are features of NF-I. These abnormalities were essentially explained by an aberrant dysfunction of the neurofibromin; a protein expressed in vascular endothelial, an anomalous in maintenance and repair of cellular components of vessel walls, a hyperproliferation of Schwann cell, pericytes and endothelial cells and an alteration of the constitution and the function of these cells [5]. These findings therefore were suggested to explain the coexisting of antiphospholipid syndrome and NF1 [6]. Referring to these issues we suggest that coexisting GAP and NF1 could be non-accidental.

In fact, GAP is the most common entity among the anti-neutrophil-cytoplasmic-antibody (ANCA) associated vasculitides (AAV) disorders and directed against proteinase 3 (PR3) antigens specifically. Its annual worldwide incidence is estimated to be 10-20 cases per one million based upon the geographical location [7]. It presents a wide spectrum of manifestations; it classically presents with upper and lower respiratory tract symptoms and renal involvement [2]. Its lung involvement, which is present in our case, is a common manifestation in children and its severity is variable, ranging from asymptomatic pulmonary lesions to dramatic life-threatening clinical presentations such as diffuse alveolar haemorrhage. Our patient was diagnosed early, only when investigated for hypereosinophilia, which is exceptional. Howewer, patients with NF1 may also present with pulmonary involvement characterized by dyspnea and tomographic evidence of interstitial lung disease, such as emphysema, bullae, cysts and reticular abnormalities [8]. In the case of our patient, we considered that his pulmonary disorder is related to the GAP because he had rather nodular lesions that appeared concurrently with nasal symptoms and hypereosinophilia. In conclusion, NF1 can be associated to several diseases, which confirms the utility of an extensive lesion assessment during the follow-up. In addition GPA should be considered while exploring hypereosinophilia as it has been reported previously in the literature [9] as it is a serious disease that can result in organs damage if not treated immediately. In our case the early diagnosis makes the better prognosis.

## Conclusion

Neurofibromatosis type 1 (NF1) can be associated to several diseases, which confirms the utility of an extensive lesion assessment during the follow-up. In addition GPA should be considered while exploring hypereosinophilia as it has been reported previously in the literature [9] as it is a serious disease that can result in organs damage if not treated immediately. In our case the early diagnosis makes the better prognosis.
